# The risk of obstructive sleep apnea is highly correlated with depressive symptoms among the Korean adults population: results from the 2020 Korea National Health and Nutrition Examination Survey

**DOI:** 10.1186/s12888-023-04983-7

**Published:** 2023-06-27

**Authors:** Mi-Sun Lee, Hooyeon Lee

**Affiliations:** Department of Preventive Medicine, College of Medicine, The Catholic University of Korea, Seoul, 06591 Korea

**Keywords:** Obstructive sleep apnea, OSA, Depressive symptom, PHQ-9

## Abstract

**Objectives:**

We aimed to examine the association between Obstructive Sleep Apnea (OSA) risk, health behaviors, and depressive symptoms in a representative Korean sample.

**Methods:**

Cross-sectional data from the 2020 Korea National Health and Nutrition Examination Survey (KNHANES) were analyzed. The sample included 4,352 adults aged 40 years and older. Multiple linear regression analysis was performed to examine the association between OSA risk, health behaviors, and depressive symptoms.

**Results:**

In total, 23.1% of the participants reported a high risk of OSA. Of the respondents, 39.8%, 19.0%, 27.2%, and 8.7% reported hypertension, snoring, tiredness, and observed apnea, respectively. The prevalence of moderate-severe depressive symptoms among adults with high-risk OSA was 7.5%. The significant associations between OSA risk and sex with PHQ-9 were shown in univariate linear regression. In the multiple linear regression analysis, the association between high risk of OSA and PHQ-9 showed in total (B = 1.58; *P* < 0.001), male (B = 1.21; *P* < 0.001), and female (B = 1.93; *P* < 0.001).

**Conclusions:**

A high risk of OSA was associated with an increased prevalence of depressive symptoms. Monitoring the risk factors of depressive symptoms, including OSA, or unhealthy behaviors may decrease the mental health issues of middle-aged and older adults.

## Introduction

Obstructive sleep apnea (OSA) is a sleep-related breathing disorder characterized by repeated episodes of pharyngeal collapse during sleep [1, 2]. Its symptoms include hypertension, excessive daytime sleepiness, snoring, observed apnea, and arousal with breathing pauses. Some adults with OSA risk also experience fatigue and excessive daytime sleepiness, which can impair cognitive functioning [3].

Its prevalence in the general population ranges from 2 to 26% depending on age, sex, and OSA criteria [4–6]. Globally, approximately 1 billion adults (aged 30–69 years) suffer from OSA, and approximately 425 million require medical treatment [7]. In the United States (U.S.), over 20 million middle-aged adults suffer from OSA. However, it was suggested that 80% of males and 93% of females with moderate-to-severe OSA remain undiagnosed [8]. In South Korea, its prevalence was 15.8% among 2,740 adults according to a nationwide questionnaire survey [9].

Several studies reported that OSA was associated with risk factors, which include the male sex, older age, family history of OSA, upper airway structural abnormalities, hyperlipidemia, obesity, alcohol consumption, smoking, and glucose intolerance [10–12]. In addition, the crucial impact of social and psychological issues on OSA-related sleep disorders has been highlighted [13]. However, despite its negative consequences and risks, mental health screening among adults with OSA remains largely underdiagnosed or undertreated in clinical settings [14].

OSA risk was also associated with depressive symptoms [15]. A previous study showed that a high risk of OSA significantly predicted the odds ratio of developing depression [16]. In addition, adults with comorbid OSA and major depression reported longer and more severe episodes of depression [17]. OSA also affects the quality of life and different aspects of health behaviors [1]. Furthermore, health behavior problems, such as drinking and smoking may exacerbate severe or chronic depressive symptoms [18–20]. The associations and relationships between OSA risk, health behaviors, and depressive symptoms, are perceived as a serious public health concern. However, our understanding of these relationships and their underlying mechanisms remain unclear [8].

Despite recent research [7, 13, 16], limited studies have examined the prevalence and risk factors of OSA among adults stratified by sex in South Korea. Therefore, this study aimed to examine the association between OSA risk, health behaviors, and depressive symptoms in a representative sample of Korean adults.

## Methods

### Study data population

We used data from the 2020 Korea National Health and Nutritional Examination Survey (KNHANES) by the Ministry of Health and Welfare [21]. The KNHANES, a cross-sectional, population-based, and continuous survey, aimed to assess the health status and trends in various chronic diseases of a representative South Korean sample [22]. KNHANES is the nationwide survey conducted every year, also, its target population is comprised of nationally representative civilians in South Korea. Additionally, this surveillance system had been conducted by the Korea Centers for Disease Control and Prevention (KCDC) [23]. The KNHANES survey was useful to monitor changes in risk factors and diseases and identify target populations that required intervention [21]. It combined health interviews with a standardized physical examination [23]. The survey obtained information from participants through face-to-face interviews. All participants in the KNHANES partook voluntarily, and informed consent was obtained. We used data from 7,359 Korean adults who responded to the 2020 survey. The current analysis was limited to 4,352 individuals aged ≥ 40 years.

### Independent variables: OSA

The STOP questionnaire (snoring, tiredness, observed apnea, and high blood pressure) was used to determine whether the respondents were at risk of OSA [4]. A previous study recommended the STOP as a screening tool for OSA in a clinical setting [24]. Snoring, tiredness, and observed apnea were assessed by yes or no responses to the questions: “Do you snore loudly (louder than talking or loud enough to be heard through closed doors)?,” “Do you often feel tired, fatigued, or sleepy during the daytime?,” and “Has anyone observed you stop breathing during your sleep?”

Information on hypertension was collected during the health examinations. Trained nurses measured systolic blood pressure (SBP) and diastolic blood pressure (DBP) via an automated device (Greenlight 300), according to standard protocols. Blood pressure was categorized into three groups: (1) normal tension (SBP < 120 mmHg or DBP < 80 mmHg), (2) pre-hypertension (120 mmHg ≤ SBP < 140 mmHg or 80 mmHg ≤ DBP < 90 mmHg), and (3) hypertension (SBP ≥ 140 mmHg or DBP ≥ 90 mmHg). Based on the STOP questionnaire responses (score range: 0–4), participants were classified into the normal (0), low-risk (1), or high-risk group (≥ 2) [10].

### Dependent variable: depressive symptoms

The Patient Health Questionnaire-9 (PHQ-9) was used to assess depressive symptoms in primary care and medical settings. It demonstrated high internal consistency, specificity, and sensitivity in identifying cases of major depressive disorder (MDD) [25, 26]. It consisted of nine items rated on a 4-point Likert scale (0–3). The total score ranged from 0 to 27, and a score of ≥ 10 represented clinically significant depressive symptoms [19]. The severity of depressive symptoms was categorized according to the total scores (0–4, 5–9, and 10–27 as normal/minimal, mild, and moderate-severe depression, respectively) [27].

### Covariate variables

Age, sex, household income, education level, and body mass index (BMI) were considered covariates. We divided participants into two groups: aged 40–64 years and ≥ 65 years. Household income level included wages, unemployment benefits, pensions, bank interests, and social security benefits [20, 21]. It was defined as the average monthly gross income divided by the equivalence factor to adjust for differences in household composition and size [20]. Household income levels were categorized into quintiles. The first quintile corresponds to the lowest income quintile and the fifth quintile is the highest income quintile [28]. Educational level was grouped into three categories: (1) college graduation or higher, (2) high school graduation, or (3) junior high school graduation or lower. Marital status was classified into two groups: (1) married or (2) others (never married, separated, divorced, or death of a spouse). Height and weight were used to calculate BMI as follows: underweight (< 18.5 kg/m^2^), normal (18.5–23 kg/m^2^), overweight (23–25 kg/m^2^), and obesity (≥ 25 kg/m^2^) [21, 23]. Current drinking and smoking experiences were categorized as yes or no.

### Statistical analysis

The complex sampling followed a multi-stage clustered design, and all statistics were calculated using weights assigned to the data sample. We performed frequency and percentage analyses stratified by sex. We compared the prevalence of depressive symptoms using a chi-squared test. A univariate linear model was evaluated to analyze the association between sex, OSA risk, and PHQ-9. The Point-biserial correlation analysis was used to examine the correlations between OSA risk and PHQ-9 by sex [29]. In addition, univariate linear regression and multiple linear regression analyses were performed to investigate the crude and adjusted linear associations between OSA risk and PHQ-9 stratified by sex. In Model 1, we presented the univariate linear regression model. In Model 2, we analyzed the multiple linear regression model adjusted for age, household income level, education level, marital status, body mass index, drinking, and smoking. Statistical analyses were performed using SPSS version 25 (IBM Corp., Armonk, NY, USA). Statistical significance was set at *p* < 0.05.

### Ethics

All participants signed an informed consent form during the KNHANES. The KNHANES study was approved by the Institutional Review Board of the Korea Centers for Disease Control and Prevention (no. 2018-01-03-2 C-A).

## Results

Table 1 presents the characteristic of study population. Of the 4,352 participants, 1,912 (43.9%) were male, and 2,440 (56.1%) were female. Among them, 78.1% were married, 66.8% currently drank, and 17.4% had smoked. Among the OSA-related variables, 39.8%, 19.0%, 27.2%, and 8.7% of participants reported hypertension, snoring, tiredness, and observed apnea, respectively. In addition, 23.1% of participants reported a high-risk of OSA. The prevalence of high-risk OSA among adults aged 65 years or older was 23.0% for males and 22.8% for females. In addition, the prevalence of high-risk OSA among adults aged younger than 65 years was 31.7% for males and 14.7% for females. The chi-squared test showed that all variables were statistically significantly different by sex (*p* < 0.001).

**Table 1:** Characteristics of the study population (*N* = 4352)

Variables	Total	Male	Female	*p-value*
	N(Weighted %)	N(Weighted %)	N(Weighted %)	
Total	4352(100.0)	1912(43.9)	2440(56.1)	
Age (years)				
40–64	2671(71.8)	1188(74.8)	1483(69.0)	< .001
≥ 65	1681(28.2)	724(25.2)	957(31.0)	
Household income level				
1^st^ quintile (lowest)	789(14.3)	291(11.4)	498(17.0)	< .001
2^nd^ quintile	873(17.6)	375(16.8)	498(18.2)	
3^rd^ quintile	835(20.3)	373(20.6)	462(20.1)	
4^th^ quintile	922(23.5)	420(24.2)	502(22.9)	
5^th^ quintile (highest)	912(24.3)	447(27.0)	465(21.8)	
Education level				
≥College	1204(35.8)	645(42.5)	559(29.5)	< .001
High school	1264(35.5)	573(35.0)	691(35.8)	
≤Middle school	1424(28.7)	500(22.5)	924(34.7)	
Marital status				
Married	3275(78.1)	1580(82.9)	1695(73.7)	< .001
Others	1077(21.9)	332(17.1)	745(26.3)	
Body mass index (kg/m^2^)				
Underweight (< 18.5)	115(2.7)	48(2.3)	67(3.1)	< .001
Normal (18.5 ≤ < 23)	1448(32.5)	500(23.5)	948(41.1)	
Overweight (23 ≤ < 25)	1049(25.6)	512(28.8)	537(22.5)	
Obesity (≥ 25)	1651(39.2)	822(45.4)	829(33.3)	
Current drinking				
Yes	2704(66.8)	1430(78.2)	1274(56.1)	< .001
No	1647(33.2)	481(21.8)	1166(43.9)	
Current smoking				
Yes	660(17.4)	569(32.2)	91(3.5)	< .001
No	3691(82.6)	1342(67.8)	2349(96.5)	
Blood pressure (mmHg)				
Normal (SBP < 120 or DBP < 80)	1328(33.4)	451(25.7)	877(40.7)	< .001
Pre-hypertension (120 ≤ SBP < 140 or 80 ≤ DBP < 90)	1098(26.7)	532(29.8)	566(23.9)	
Hypertension (SBP ≥ 140 or DBP ≥ 90)	1845(39.8)	890(44.5)	955(35.5)	
Snoring				
Yes	769(19.0)	454(25.9)	315(12.4)	< .001
No	3582(81.0)	1457(74.1)	2125(87.6)	
Tiredness				
Yes	1156(27.2)	473(25.9)	683(28.3)	.015
No	3195(72.8)	1438(74.1)	1757(71.7)	
Observed apnea				
Yes	342(8.7)	261(14.3)	81(3.5)	< .001
No	4009(91.3)	1650(85.7)	2359(96.5)	
STOP (0–4)				
Normal (0)	1483(36.3)	555(30.6)	928(41.7)	< .001
Low risk of OSA (1)	1814(40.6)	790(39.9)	1024(41.1)	
High risk of OSA (≥ 2)	974(23.1)	528(29.5)	446(17.2)	
PHQ-9 (0–27)				
M(SD)	2.26(3.47)	1.71(3.09)	2.51(3.70)	< .001
Normal/minimal (0–4)	3241(84.6)	1509(88.0)	1732(81.4)	
Mild (5–9)	434(11.4)	140(8.6)	294(14.0)	
Moderate-severe (≥ 10)	176(4.0)	56(3.4)	120(4.7)	

*DBP* Diastolic blood pressure, *M* Mean, *OSA* Obstructive Sleep Apnea, *SBP* Systolic blood pressure, *SD* Standard deviation, *STOP* Snoring, tiredness, observed apnea, and high blood pressure, *PHQ-9* Patient Health Questionnaire-9

Table 2 shows the prevalence of depressive symptoms among the Korean population aged 40 years and older. The prevalence of mild and moderate-to-severe depressive symptoms was 11.4% and 4.0%, respectively. Moderate-to-severe depressive symptoms were 4.7% in females and 3.4% in males. Among the lowest household income level, the prevalence of moderate-to-severe depressive symptoms was 11.1%. In addition, hypertension, snoring, tiredness, and OSA were risk factors for depressive symptoms. The prevalence of moderate-severe depressive symptoms among adults with high-risk OSA was 7.5%.

**Table 2:** Prevalence of depressive symptoms in Korean adults aged 40 years and older (weighted %)

Variables	Depressive symptoms (PHQ-9)			*p-value*
	Normal/minimal (0–4)	Mild (5–9)	Moderate-severe (≥ 10)	
Total	84.6	11.4	4.0	
Sex				
Male	88.0	8.6	3.4	< .001
Female	81.4	14.0	4.7	
Age (years)				
40–64	84.7	11.5	3.8	.171
≥ 65	84.1	11.0	4.9	
Household income level				
1^st^ quintile (lowest)	72.2	16.6	11.1	< .001
2^nd^ quintile	84.5	11.9	3.6	
3^rd^ quintile	83.1	12.9	4.0	
4^th^ quintile	84.9	11.5	3.6	
5^th^ quintile (highest)	91.5	7.2	1.3	
Education level				
≥College	89.1	8.5	2.4	< .001
High school	82.5	13.2	4.2	
≤Middle school	81.5	12.7	5.9	
Marital status				
Married	86.9	10.4	2.7	< .001
Others	75.6	15.3	9.1	
Body mass index (kg/m^2^)				
Underweight (< 18.5)	80.8	15.0	4.2	.122
Normal (18.5 ≤ < 23)	83.6	12.4	4.0	
Overweight (23 ≤ < 25)	86.1	9.9	4.0	
Obesity (≥ 25)	85.2	11.1	3.7	
Current drinking				
Yes	85.0	11.4	3.6	.160
No	83.8	11.2	5.0	
Current smoking				
Yes	78.9	13.9	7.3	< .001
No	85.8	10.8	3.3	
Blood pressure (mmHg)				
Normal tension (SBP < 120 or DBP < 80)	83.9	12.2	3.9	.114
Pre-hypertension (120 ≤ SBP < 140 or 80 ≤ DBP < 90)	87.3	9.4	3.4	
Hypertension (SBP ≥ 140 or DBP ≥ 90)	83.4	12.0	4.6	
Snoring				
Yes	81.6	14.1	4.3	.075
No	85.4	10.6	4.0	
Tiredness				
Yes	69.6	20.7	9.8	< .001
No	91.0	7.4	1.6	
Observed apnea				
Yes	81.1	15.0	3.9	.059
No	85.0	11.0	4.1	
STOP (0–4)				
Normal (0)	91.6	6.8	1.7	< .001
Low risk of OSA (1)	83.9	12.2	3.9	
High risk of OSA (≥ 2)	76.0	16.4	7.5	

*DBP* Diastolic blood pressure, *OSA* Obstructive Sleep Apnea, *PHQ-9* Patient Health Questionnaire-9, *SBP* Systolic blood pressure, *STOP* Snoring, tiredness, observed apnea, and high blood pressure

**Fig. 1:**
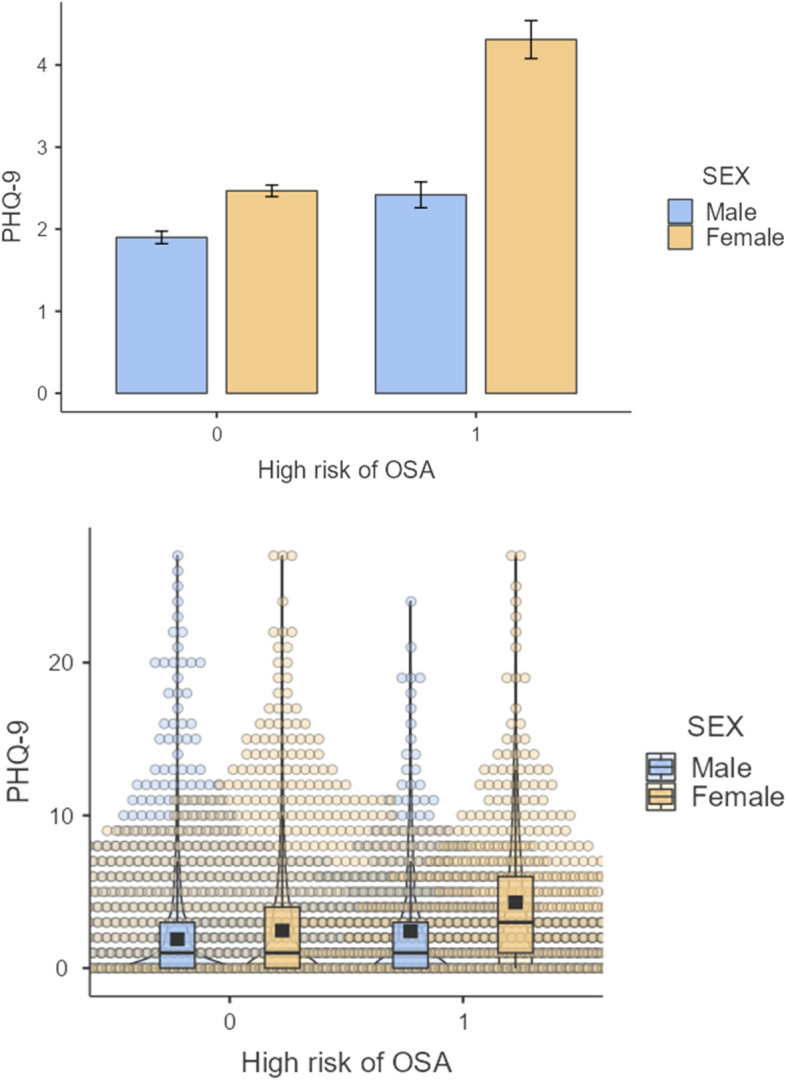
Distribution and Density plot of the relationship between PHQ-9 scores and high-risk OSA by sex. *Note.* OSA: Obstructive Sleep Apnea, PHQ-9: Patient Health Questionnaire-9

Figure 1 lists the distribution and Density plot of the relationship between PHQ-9 and high-risk OSA by sex was visualized and presented.

Table 3 shows the Point-biserial correlation analyses for associations between OSA risk and PHQ-9 stratified by sex. In both sexes, OSA-related indicators (hypertension, snoring, tiredness, and observed apnea) showed a statistically significant and positive correlation with the PHQ-9 (all *p*-value < 0.05).

**Table 3:** Point-biserial correlations between OSA risk and PHQ-9^a^

	Male		Female	
	Correlation coefficient	*p-value*	Correlation coefficient	*p-value*
Hypertension	.042	.009	.056	.001
Snoring	.061	< .001	.033	.007
Tiredness	.056	< .001	.067	< .001
Observed apnea	.031	.011	.058	.019

*OSA* Obstructive Sleep Apnea, *PHQ-9* Patient Health Questionnaire-9

^a^A point-biserial correlation was used to measure the strength and direction of the association between the continuous variable and the dichotomous variable

Table 4 represents the univariate linear regression and multiple linear regression analyses for associations between OSA risk and PHQ-9 stratified by sex. The significant associations between OSA risk and sex with PHQ-9 were shown in multiple linear regression (all *p*-value < 0.001). In the results of multiple linear regression analysis after adjusting the covariates (age, household income level, education level, marital status, body mass index, drinking, and smoking), the association between high risk of OSA and PHQ-9 remained in the total (B = 1.58; *P* < 0.001), male (B = 1.21; *P* < 0.001), and female (B = 1.93; *P* < 0.001). Additionally, the influence of sex on the PHQ-9 was also statistically significant (*P* < 0.001).

**Table 4:** Sex-specific linear regression analysis for associations between OSA risk and PHQ-9

	Model 1			Model 2		
	B	S.E.	*p-value*	B	S.E.	*p-value*
All						
Sex^a^	1.37	.07	< .001	1.19	.05	< .001
High risk of OSA	2.20	.11	< .001	1.58	.06	< .001
Male						
High risk of OSA	1.79	.09	< .001	1.21	.07	< .001
Female						
High risk of OSA	2.82	.15	< .001	1.93	.10	< .001

Model 1: univariate linear regression model

Model 2: multiple linear regression model adjusted for age, household income level, education level, marital status, body mass index, drinking, and smoking

*OSA* Obstructive Sleep Apnea, *PHQ-9* Patient Health Questionnaire-9, *S.E.* Standard error

^a^‘0’ for male and ‘1’ for female in the analysis

## Discussion

In this study, the prevalence of high-risk OSA was 23.1% among Korean adults aged ≥ 40 years. This finding was similar to that of a previous Norwegian population-based study that reported a 24.3% prevalence [30]. Globally, the prevalence of OSA ranges from 2 to 26% [4]. In contrast, data from a 2010 South Korean population study showed a prevalence of 15.8% [9], which was lower than that reported in the current study. A prior study used the Berlin Questionnaire (BQ) to assess OSA. However, the STOP questionnaire had a higher sensitivity and was indicated to be more suitable for discriminating Korean adults with moderate-to-severe OSA [31]. Thus, the differences in the screening tools among the study populations may be the reason for the differences in its prevalence. Furthermore, its prevalence varies with diagnostic criteria, measurement methods, and apnea-hypopnea index cut points [9]. Overnight polysomnography (PSG) is the standard clinical examination for diagnosing OSA [32]. However, STOP is widely used as a measure in asymptomatic populations at the community level [33]. Therefore, a brief screening tool could assist adults with OSA in the community or general population.

In this study, the prevalence of high-risk OSA was higher in males (29.5%) than in females (17.2%). A previous study reported that the prevalence was 1.83 times higher in males than that in females in the South Korean population [9]. A previous epidemiologic study also reported that males had a higher prevalence of OSA than females [34]. A population-based study reported that 24–47% of males and 9–30% of females had sleep-disordered breathing [35]. In contrast, sex differences in OSA decreased with increasing age [36]. In the current study, the prevalence of high-risk OSA (male 23.0%, female 22.8%) in adults aged 65 years or older showed a lower sex difference compared to adults aged younger than 65 years (male 31.7%, female 14.7%). Another study also indicated that high-risk OSA in postmenopausal women increased more than that in men [2]. Therefore, attention should be paid to the age and physical symptoms or changes, as well as the sex of middle-aged or older adults at risk of OSA.

Our study showed that the high-risk groups for OSA were highly associated with moderate-to-severe depressive symptoms. This finding was consistent with the previous results that OSA was associated with depressive symptoms [13, 17, 37]. There was evidence that adults with a high risk for OSA had increased rates of depressive symptoms. A longitudinal population-based study showed that OSA severity significantly predicted the odds of developing depressive symptoms [15]. Specifically, a prior longitudinal study also identified OSA as an independent risk factor for depression, and the OR of developing depressive symptoms increased 2.0 and 2.6 times among participants with mild and moderate-to-severe OSA, respectively [37]. An understanding of OSA and the risk factors for depressive symptoms could lead to its prevention or treatment [1]. Adults with a high risk of OSA may visit psychiatric clinics with complaints, such as daytime sleepiness, fatigue, an increase in irritability or agitation, sleep disturbances, and chronic depressive mood [38]. Therefore, in middle-aged and older adults with OSA risk, clinicians should routinely screen for and inquire about depressive symptoms.

The current study showed that the level of depressive symptoms increased with OSA risk in both sexes, also, the trend was more noticeable in females than males. The high risk of OSA indicated positive correlations with depressive symptoms with larger regression coefficients in females. These findings could provide the associations between OSA risk and depressive symptoms in the general Korean adult population. Similarly, recent findings also demonstrated that OSA risk was associated with depressive symptoms among females [6, 9, 13, 16]. Thus, our data suggest that more control and screening of the potential depressive symptoms may be needed in women at high risk of OSA.

This study has several limitations. First, it used cross-sectional data. Therefore, the results of causation were precluded by the possibility of reverse causality. Second, since the STOP questionnaires, except for blood pressure measurements, were examined based on self-reports, they may be less accurate than those measured by clinical examinations. However, the prevalence of OSA risk in the Korean population-based data was a significant finding. Additionally, the severity of depressive symptoms was determined using a valid and reliable standardized tool [26].

## Conclusions

In conclusion, many middle-aged and older adults may have OSA risk for several years; however, they remain undiagnosed. Our findings are the first to evaluate the association between OSA risk, health behaviors, and depressive symptoms in a representative sample of Korean adults. This study suggests that future preventions and interventions should include primary efforts, such as mental health assessment and psychoeducation, targeted at high-risk OSA among adults aged 40 years or older. Accordingly, early evaluation and treatment could prevent chronic depression and mental health illnesses.

## Data Availability

Original data are publicly available for free from the KNHANES website (https://knhanes.kdca.go.kr/knhanes/sub04/sub04_04_01.do) for purposes such as academic research. We used the SPSS dataset of KNHANES in 2020. The authors do not possess the right to directly distribute the data.
